# Rethinking Heart Failure

**DOI:** 10.4021/cr228w

**Published:** 2012-11-20

**Authors:** Hauke Fürstenwerth

**Affiliations:** Dr. Hauke Fürstenwerth, Unterölbach 3A, D-51381 Leverkusen, Germany. Email: hauke@fuerstenwerth.com

**Keywords:** Heart failure, Catecholamine, Metabolism, Autonomous nervous system, Ouabain, Digoxin

## Abstract

An increasing body of clinical observations and experimental evidence suggests that cardiac dysfunction results from autonomic dysregulation of the contractile output of the heart. Excessive activation of the sympathetic nervous system and a decrease in parasympathetic tone are associated with increased mortality. Elevated levels of circulating catecholamines closely correlate with the severity and poor prognosis in heart failure. Sympathetic over-stimulation causes increased levels of catecholamines, which induce excessive aerobic metabolism leading to excessive cardiac oxygen consumption. Resulting impaired mitochondrial function causes acidosis, which results in reduction in blood flow by impairment of contractility. To the extent that the excessive aerobic metabolism resulting from adrenergic stimulation comes to a halt the energy deficit has to be compensated for by anaerobic metabolism. Glucose and glycogen become the essential nutrients. Beta-adrenergic blockade is used successfully to decrease hyperadrenergic drive. Neurohumoral antagonists block adrenergic over-stimulation but do not provide the heart with fuel for compensatory anaerobic metabolism. The endogenous hormone ouabain reduces catecholamine levels in healthy volunteers, promotes the secretion of insulin, induces release of acetylcholine from synaptosomes and potentiates the stimulation of glucose metabolism by insulin and acetylcholine. Ouabain stimulates glycogen synthesis and increases lactate utilisation by the myocardium. Decades of clinical experience with ouabain confirm the cardioprotective effects of this endogenous hormone. The so far neglected sympatholytic and vagotonic effects of ouabain on myocardial metabolism clearly make a clinical re-evaluation of this endogenous hormone necessary. Clinical studies with ouabain that correspond to current standards are warranted.

## Rethinking Heart Failure

The heart is the sovereign of the body. It drives the circulation, distributes vital nutrients and neurotransmitters via the blood stream and provides substance- and information-exchange between different organs. The heart is not consciously controlled; it is subject to control by the autonomous nervous system. Unlike skeletal muscles, there are no phases of rest for the heart muscle, which would allow recovery. The performance of the heart is brought about by its contractions. Its adjustment to changing performance demands is done so only through change in contractile output: change in contractile force, stroke rate, stroke volume and increase in size of the ventricles (hypertrophy). The pumping output varies widely. The myocardial blood flow ranges from as low as 0.3 mL/minute/g to as high as 5 - 6 mL/minute/g.

The heart not only provides adequate perfusion to every organ of the body but also is itself dependent on constant energy supply via the blood stream. The perfusion of the heart is secured by innate compensatory mechanisms. Coronary artery occlusion can result in development of an effective intercoronary collateral circulation. If occlusion proceeds gradually, sufficient collateral vessel recruitment and growth may occur to allow progression to total arterial occlusion with little or no infarction of the dependent myocardium. Collaterals and anastomoses allow sufficient blood flow even in cases with total coronary occlusion [1]. Extreme cases are reported where total occlusion of all three major coronary arteries still allowed sufficient blood flow and normal heart function. In addition, extracardiac anastomoses support perfusion of the heart [2].

Effective myocardial function requires continuous energy provision. The high energy requirements of the myocardium are fulfilled by high rates of adenosine triphosphate (ATP) synthesis and hydrolysis. There is a nearly complete turnover of the myocardial ATP pool every few seconds, with the heart cycling approximately 6 kg of ATP per day. A high degree of metabolic flexibility guarantees these high energy demands. The heart is a metabolic omnivore. It utilizes various energy substrates including fatty acids, glucose, lactate, ketone bodies and even some amino acids to generate ATP. A substantial amount of ATP production is attributable to mitochondrial oxidative phosphorylation. Mitochondria occupy about 30% of the volume of a cardiac myocyte, ensuring the great oxidative capacity of the system.

An important substrate for the heart is oxygen. At a heart rate of 60 - 70 beats/min for the human heart the oxygen consumption normalized per gram of myocardium is 20-fold higher than that of skeletal muscle at rest. The heart achieves a very high level of oxygen extraction of 70 - 80% compared with 30 - 40% in skeletal muscle. This is ensured by a capillary density of 3,000 - 4,000/mm^2^, compared to 500 - 2,000 capillaries/mm^2^ in skeletal muscle. The human heart contains an estimated 2 - 3 billion cardiac muscle cells. But these account only for less than a third of the total cell number in the heart. The total includes a broad array of additional cell types. The distinct cell pools are not isolated from one another within the heart, but instead interact physically and via cellular crosstalk by a variety of transmitters. The heart still holds a lot of mysteries that have yet to be deciphered.

## Oxidative Capacity is Reduced in Heart Failure

Heart failure (HF) has emerged as the leading cause of morbidity and mortality in developed countries. The five-year mortality from heart failure is over 50% and equals the one from cancer [3]. HF is clinically defined as a complex clinical syndrome that can result from any structural or functional cardiac disorder that impairs the ability of the ventricle to fill with or eject blood. HF is a syndrome characterized initially by left ventricular dysfunction. Left ventricular (LV) dysfunction represents the final common pathway for most forms of heart disease. LV dysfunction is commonly attributed to myocardial ischemia. Myocardial ischemia and its usual manifestations, angina pectoris and acute coronary syndrome, today are classified as the result of an imbalance between myocardial oxygen supply and myocardial oxygen demand. Ischemia is a local phenomenon that can experimentally be produced by reducing or eliminating the flow of coronary arterial blood to a localized area of myocardium by obstructing the coronary artery supplying this region. Thus it differs from “hypoxia”, which describes a pathological condition in which the body as a whole or an organ like the heart is deprived of adequate oxygen supply without limitation in blood flow. The effects of ischemia are usually more severe than hypoxia and typically include acidosis, diminished mitochondrial energy production and cell death. The term “ischemia” was first used by Rudolf Virchow in 1858: “so habe ich den neuen Ausdruck der Ischaemie vorgeschlagen, um damit die Hemmung der Blutzufuhr, die Vermehrung der Widerstande des Einstromens zu bezeichnen” [4]. Virchow’s definition of “ischemia” only refers to inhibition of the blood supply that is caused by an increased resistance to the blood flow. Today the term “ischemia” is commonly used in a broader sense, meaning that the blood supply to the myocardium is inadequate to maintain normal oxygen demand. High-grade coronary stenosis and rupture of vulnerable plaques are held responsible as the usual cause of such decreased oxygen supply.

Thus the basic concept in heart diseases is that these primarily result from a deficit in oxygen supply to the heart. Invasive therapies - percutaneous transluminal coronary angioplasty and coronary artery bypass graft surgery - as well as many medications (anticoagulants, thrombolytic agents, platelet aggregation inhibitors, statins) are based on that paradigm. Although the dogma of insufficient oxygen supply due to limitations in blood flow today is generally accepted, critics always have pointed out findings, which are incompatible with this model. Alternative concepts have been presented, proposing that coronary heart disease is more an adrenergic stress-dependent disease than a hydraulic problem [5, 6]. Clinical experience supports doubts as to the general validity of oxygen deficiency as the central cause of heart disease. Invasive treatments yield only marginal effects on mortality and have failed to show any incremental clinical benefit compared to medical therapy for the reduction of death or nonfatal myocardial infarction [7-9]. The efficacy of cholesterol lowering drugs in prevention of myocardial infarctions is disputed. Due to various side effects, the FDA on February 28, 2012 has issued a new warning label for all statin drugs.

Only the identification of neurohumoral activation as a central detrimental feature of HF and subsequent development of neurohumoral antagonists (angiotensin converting enzyme inhibitors, angiotensin II receptor antagonists, β-adrenergic receptor antagonists, aldosterone receptor antagonists) has led to great advances in the treatment of HF. Nonetheless, the residual disability and death rate remains unacceptably high. HF is becoming epidemic as the population ages [10].

One characteristic feature for all forms of heart failure is changes in myocardial energy metabolism. Profound abnormalities have been identified in heart failure, that correlate with clinical symptoms and survival. There is evidence that the myocardium in HF is energy-depleted. Myocardial ATP levels are progressively reduced by approximately 25-35%, creatine kinase activity and total creatine pool are decreased by as much as 50-70% in the failing heart [11]. Based on the concept of limited oxygen supply, strategies have been designed to shift myocardial metabolism away from a preference for fatty acids towards more carbohydrate oxidation [12]. Such a shift is expected to result in a relatively greater production of ATP per unit of oxygen consumed. This concept is supported by the fact that glucose in the foetal heart is the preferred substrate and the finding that in advanced stages of heart failure cardiac fatty oxidation is reduced and glycolysis and glucose oxidation are increased. However, clinical trials based on metabolic modulators have been disappointing and have not yet resulted in widely applicable drugs [13].

An increasing body of evidence sheds new light on metabolism in ischemia-induced heart diseases. Research on myocardial metabolism has shown that oxygen is not limiting in the failing myocardium. The failing heart is a unique example of a well-oxygenated heart. Studies in several animal models indicate that inadequate oxygen supply or demand ischemia does not contribute significantly to heart failure progression [14]. Instead oxidative capacity and function are reduced in the failing myocardium. Decreased capacity of mitochondrial substrate oxidation leads to decreased cardiac efficiency [15]. In response to rapid pacing, the failing canine heart is unable to significantly increase its oxidation of fatty acids or glucose, as was the normal myocardium [16]. These observations are supported by human data [17]. Oxidative capacity is reduced in human and experimental heart failure [18]. Patients with idiopathic dilated cardiomyopathy show a failure to increase glucose uptake and possibly oxidative glycolysis in response to pacing stress [19]. In failing hearts the ability to oxidize both fatty acids and glucose is impaired. The risk of heart disease increases with age. Elderly subjects have nearly 50 % lower oxidative capacity per volume of muscle than adult subjects due to reduction in mitochondrial content, as well as a lower oxidative capacity of the mitochondria [20]. Obviously it is not a limited availability of metabolic substrates or oxygen, but the impaired ability to consume the available substrates that characterizes the failing heart [21]. That is why Taegtmeyer’s question “Why does the heart fail in the midst of plenty?’’ [22] is still valid, and also still remains unanswered.

## Acidosis and Not Hypoxia or Ischemia Triggers Cell Death

According to standard interpretation, ischemia leads to anaerobic glycolysis, which results in a progressive accumulation of protons, inorganic phosphate, sodium, and calcium, ultimately severely curtailing synthesis of ATP. The accumulation of inorganic phosphate and of protons has been implicated as a causal link to decreased contractile performance, whereas the accumulation of calcium in mitochondria and cytosol has been implicated as a causal link to irreversible cell damage [23]. Acidosis, and not hypoxia or ischemia alone is a major trigger of apoptosis and cell death in cultured cells and cardiac myocytes. Living atrial human tissue, when subjected to acidosis, exhibits apoptotic changes proportional to the degree of acidosis [24]. In addition, a decreasing intracellular pH inhibits insulin-signalling [25]. Without energy supply the cellular ion-transport system collapses. Thus prolonged ischemia will induce progressive failure of the ionic homeostasis and further decline in the ATP stores, which will eventually cause ischemic contracture. However, experiments have shown that the heart can survive for minutes, if not hours, with only limited supply of oxygen.

In 1966 Hochrein investigated the effects of hypoxia in guinea pig heart-lung preparations [26]. On nitrogen breathing hypoxic heart failure develops. Simultaneous infusion of glucose, insulin, and potassium salts under continued nitrogen breathing stopped progression of heart failure and resulted in “a nearly complete disappearance of hypoxic failure”. Despite total absence of oxygen the guinea pig heart recovered and survived.

In 1992 Webster and co-workers reported that rapidly contracting cardiac myocytes remain fully viable and contractile during culture under severe hypoxia for up to 5 days [27]. In a follow-up study cardiac myocytes were subjected to severe hypoxia for a week under conditions where the glucose and extracellular pH were constantly monitored and maintained within the physiological range [28]. These experiments revealed that cardiac myocytes are resistant to chronic hypoxia at neutral pH but undergo extensive death when the extracellular pH drops below 6.5. Obviously chronic severe hypoxia is not necessarily a lethal stress for cardiac myocytes. It is the secondary effects of hypoxia including energy depletion and metabolite accumulation (acidosis) that impose lethal effects. To remain viable under hypoxia, the cells must be able to maintain glycolysis at a level that is sufficient to sustain ATP, a condition that requires a continuous supply of glucose. In addition, the cell must be able to clear excess acid produced under hypoxia. If these conditions can not be fully accommodated, the cell will die. Acidosis, too, is responsible for chest pain commonly observed in angina pectoris. Specific acid-sensing ion channels trigger anginoid chest pain [29].

Since acidity caused by hypoxia leads to derangement of ionic homeostasis and subsequently to cell death it would stand to reason to block the Na^+^/H^+^ exchanger (NHE). NHE is a protein that is expressed in many mammalian cell types. It is responsible for intracellular pH and cell volume regulation by extruding protons from, and taking up sodium ions into cells. The NHE is quiescent at physiological values of intracellular pH but becomes activated in response to acidosis and causes intracellular accumulation of Na^+^, which leads to intracellular Ca^2+^ accumulation (Ca^2+^ overload) and myocardial injury. Hence NHE inhibitors were expected to afford a cardioprotective effect through the limitation of Na^+^ influx during ischemia and to diminish excitability and necrosis. Extensive pre-clinical work indicated that inhibition of NHE affords significant protection to myocardium subjected to ischemia. However, clinical studies with NHE inhibitors have provided largely disappointing results [30].

## Acidosis Results From Excessive Aerobic Metabolism

The secondary consequences of hypoxia, including energy depletion and acidosis rather than hypoxia per se cause cell death. Hence the origin of protons that cause acidosis is of particular importance. In situations of limited oxygen supply, like hypoxia and ischemia, the heart switches to anaerobic energy production. Glucose and glycogen become the essential nutrients. Glycogen serves as substrate reservoir to buffer rapid increases in energy demand. Glycogen stores in the heart have been linked to increased survival in the state of anoxia [31]. Glycogen stores are markedly increased in hibernating myocardium. The effect of glycogen on resistance to ischemia is most dramatic in turtle heart muscle, which is particularly rich in this compound. Seal cardiomyocytes also tolerate low oxygen conditions better than rat cardiomyocytes [32]. Studies indicate that glycogen also contributes significantly to aerobic myocardial glucose use. Glucose derived from glycogen is oxidized preferentially compared with exogenous glucose [33]. In isolated perfused working rat hearts stimulation of glycogen synthesis repartitions glucose-6-phosphate away from the glycolytic pathway. The reduced rate of glycolysis lessens H^+^ production from glucose metabolism and subsequently reduces Ca^2+^ overload [34].

It is commonly stated that anaerobic metabolism results in formation of lactic acid. Lactic acid, measured as lactate, is considered as a marker of tissue hypoxia and anaerobic metabolism. However, research has uncovered that lactate can no longer be considered the metabolic dead-end waste product of glycolysis due to oxygen deficits, but is instead a central player in cellular, regional and whole body metabolism [35, 36]. Lactate formation and its subsequent distribution throughout the body is a major mechanism whereby the coordination of intermediary metabolism in different tissues can be accomplished. Comparative examination of the glycolytic pathway across the animal kingdom has provided evidence that anaerobic conditions are not essential for lactate to be produced, confirming the dissociation between lactate and hypoxic or anoxic conditions. The widespread assessment that lactate production indicates oxygen lack is inconsistent with observations that lactate is formed and utilized continuously in diverse cells under fully aerobic conditions [37].

Blood glucose and glycogen reserves in diverse tissues can be mobilized to provide lactate, which can either be used within the cells of formation or transported to adjacent and anatomically distributed cells for utilization. Lactate is an important oxidizable substrate and gluconeogenic precursor as well as a means by which metabolism in diverse tissues is coordinated. In vivo, lactate is a preferred substrate and high blood lactate levels down-regulate the use of glucose and free fatty acids. Since lactate is released into the systemic circulation and taken up by distal tissues and organs, lactate may be an important signalling molecule, for which Brooks has suggested the term ‘lactormone’ [37].

Available data indicate that lactate is not harmful. Many studies have infused animals or humans with exogenous lactate, demonstrating its safety and usefulness. Even the brain can take up lactate from the blood. Lactate is a major energy substrate for the brain [38]. Lactate improves cardiac function in a model of hemorrhagic shock [39], increases cardiac output in postoperative patients [40] and also in cardiogenic shock [41].

The heart is an active lactate consumer. Experimental evidence suggests that as blood lactate concentration, myocardial blood flow, and myocardial oxygen consumed per minute increase, lactate becomes the preferred fuel for the heart, accounting for as much as 60% of the substrate used by human myocardium [35, 42]. Tracer studies indicate that essentially all of the lactate taken up by the heart is oxidized as an aerobic fuel [43].

Another widespread misconception is that the anaerobic production of lactate is equivalent to the production of lactic acid. It is commonly asserted that under anaerobic conditions, glycolysis results in “lactic acidosis”. Robergs et al. reviewed evidence that there is no biochemical support for lactate production causing all of the intracellular acidosis [43]. Lactate production actually retards acidosis, whilst acidification results from other biochemical processes such as ATP breakdown and the earlier stages of glycolysis. Acidosis is not caused by lactate production. Or as Robergs et al point out: “The lactic acidosis explanation of metabolic acidosis is not supported by fundamental biochemistry, has no research base of support, and remains a negative trait of all clinical, basic, and applied science fields and professions that still accept this construct. Nevertheless, statements that imply that “lactic acid” or a “lactic acidosis” causes metabolic acidosis can still be found in the current literature and remains an explanation for metabolic acidosis in current textbooks of biochemistry, exercise physiology, and acid-base physiology. Clearly, academics, researchers, and students of the basic and applied sciences, including the medical specialties, need to reassess their understanding of the biochemistry of metabolic acidosis” [43].

The biochemical processes that produce and consume protons in myocardial ischemia have been outlined in detail [44]. Taking into account all relevant processes, it can be reasoned that ischemic acidosis is predominantly due to retention of protons from glycolytic ATP turnover, carbon dioxide accumulation and net ATP breakdown, confirming that acidosis is not the result of lactate formation. Hydrolysis of ATP is the major source for protons under anaerobic as well as under aerobic conditions. When ATP is resynthesized under aerobic conditions by oxidative phosphorylation, the protons produced by ATP hydrolysis are reused in mitochondrial respiration. However, when ATP is resynthesized under anaerobic conditions by glycolysis, the protons produced by ATP hydrolysis are not reused but accumulate and contribute to acidosis. In addition, since ischemic tissue is not totally devoid of oxygen, some carbon dioxide producing oxidative decarboxylation of substrates -“respiratory acidosis” - still occurs and enhances acidosis. Proton accumulation leads to impaired contractility that results in reduced blood flow in the ischemic tissue and reduced washout of protons. Poor flow in ischemic tissue hence contributes significantly to acidosis.

The rate of lactate production is not involved in the development of acidosis. The reduction of pyruvate to lactate actually consumes protons and thus is an alkalinizing reaction that buffers acid production from glycolysis [43]. Note that the resulting acidity from the biochemical processes in both intracellular and extracellular fluids depends on the overall buffering capacities; also, the underlying reaction equilibria are pH sensitive, for details see [45].

It is well documented that “lactic acidosis” not only results from hypoxia but is seen in a variety of circumstances as a response to inflammatory mediators, catecholamines, and other factors stimulating Na^+^/K^+^-ATPase activity, classically referred to as “type B lactic acidosis”. This is of special importance in sepsis and shock, where acid accumulation can occur, despite adequate oxygen delivery [46]. Hypermetabolic conditions are observed in shock states like septic shock that are characterized by insulin resistance, hyperlactatemia, and increased oxygen demand, resulting from both enhanced mitochondrial oxygen utilization and oxygen radical production, which may coincide both with compromised tissue microcirculatory perfusion and mitochondrial dysfunction [47].

Similar to the case with heart disease, acidity causes the deterioration of additional disease states. In patients with severe sepsis or septic shock, acidosis not hyperlactatemia was found to predict in-hospital mortality more exactly [48]. Acidosis results from aerobic glycolysis, when the rate of glucose metabolism exceeds the oxidative capacity of the mitochondria [49]. In skeletal muscle and other tissues, aerobic glycolysis is linked to ATP provision for the Na^+^-K^+^ pump, the activity of which is stimulated by epinephrine.

## Catecholamines Induce Mitochondrial Dysfunction

Autonomous regulation of the heart results from a complex interaction of neurohumoral mechanisms. Of special importance are the sympathetic nervous system and its functional antagonist the parasympathetic nervous system [50]. There is a close relationship between cardiac dysfunction and autonomic dysregulation during the development of HF [51]. In patients with acute coronary syndrome attacks of angina pectoris are ordinarily triggered by sympathetic-stimulating, catecholamine-liberating conditions (exercise, emotions and other stresses), and prevented by antiadrenergic measures. Catecholamines at low concentration are beneficial in regulating heart function by exerting a positive inotropic action on the myocardium, and by acceleration of glucose uptake and oxidation; whereas high concentrations of catecholamines or chronic exposure to catecholamines over a prolonged period produce deleterious effects on the cardiovascular system. Excessive release of catecholamines induces myocardial hypertrophy, myocyte damage and contractile dysfunction resulting in infarct-like necrosis of the heart muscle. Levels of circulating catecholamines closely correlate with the severity and poor prognosis in heart failure and are thus considered to play a critical role in the development of cardiovascular diseases [52].

Most patients with heart failure demonstrate an excessive activation of the sympathetic nervous system and a decrease in parasympathetic tone at rest or during exercise. Patients with unstable ischemic symptoms have increased cardiac sympathetic nervous activity compared to patients with stable angina. Data indicate that changes in vagus nerve control of heart rate become apparent at a very early developmental stage of LV dysfunction. This autonomic dysregulation is associated with increased mortality. Muscle sympathetic neural activity (MSNA) increases with age, on average by approximately one burst per minute each year. Among patients with heart failure, MSNA is significantly increased. A healthy person on average experiences 30 to 50 sympathetic bursts per 100 heartbeats, whereas patients with heart failure can experience as many as 90 to 100 bursts per 100 heartbeats [53]. This extreme sympathoexcitation with one burst in every cardiac cycle is a predictor of mortality for patients with heart failure [54]. Unstable angina and acute myocardial infarction are associated with an increase in MSNA that lasts for several months [53].

In the human cardiovascular system norepinephrine is the primary neurotransmitter. Increased plasma norepinephrine levels, central sympathetic outflow, and norepinephrine plasma spillover from activated sympathetic nerve fibers are characteristics of sympathetic hyperactivity. Increased plasma norepinephrine levels in patients with HF are closely related to the severity of the HF, with very high norepinephrine levels found in advanced untreated HF patients [55]. LV dysfunction has been reported in the case of endogenous over-production of catecholamines in patients with pheochromocytoma [56] or severe brain injury [57]. The degree of sympathetic activation is a major and independent determinant of the myocardial, cerebral, or cardiac disease prognosis [52].

Chronic activation of the sympathetic nervous system is associated with components of the metabolic syndrome, such as blood pressure elevation, obesity, dyslipidemia, and impaired fasting glucose with hyperinsulinemia. Adrenergic stimulation leads to increased oxygen consumption. Under physiological conditions catecholamines induce an enhanced rate of aerobic glycolysis with enhanced ATP production and glucose release, both from glycogenolysis and gluconeogenesis, as well as inhibition of insulin-mediated glycogenesis [47]. Catecholamines accelerate aerobic glycolysis by stimulation of Na^+^/K^+^ ATPase, thereby generating ADP and protons [35]. In skeletal muscle epinephrine causes a dose-dependent stimulation of Na^+^K^+^-ATPase resulting in drastically enhanced aerobic lactate production [58]. In isolated working rat hearts epinephrine increased dramatically glycolysis and glucose oxidation. Epinephrine increases the uncoupling between glycolysis and glucose oxidation, which results in a significant increase in H^+^ production from glucose metabolism [59]. A disproportionate stimulation of glycolysis relative to glucose oxidation not only contributes to intracellular acidosis but may also attenuate the cardioprotective effects of insulin [60]. In a dog model of chronic HF, increased plasma norepinephrine leads to increased levels of free fatty acids (FFA), insulin, and glucose [61]. Catecholamines can decrease the viability of cardiomyocytes through cyclic AMP-mediated calcium overload and oxygen-derived free radicals [62]. Catecholamines cause mitochondrial dysfunction. Elevated FFA act on mitochondria with formation of reactive oxygen species, which in consequence results in mitochondrial and cellular dysfunction [63]. Catecholamine-stimulated triglyceride lipolysis and fatty acid activation is an additional important source for generation of protons [44] and hence contributes significantly to acidosis when oxidative phosphorylation of ADP is impaired.

## Sympathetic Over-Stimulation Results in Heart Failure

The evidence referred to suggests that heart failure is caused by sympathetic over-stimulation of cardiac metabolism. Oxygen is not limiting in the failing myocardium. Patients with heart failure demonstrate an excessive activation of the sympathetic nervous system. In heart failure the oxidative capacity of the myocardium is impaired. Sympathetic activation accelerates aerobic glycolysis, increases levels of free fatty acids, insulin, and glucose (induced insulin resistance) and stimulates triglyceride lipolysis and fatty acid activation and in consequence leads to acidosis. Acidosis inhibits synthesis of ATP. Decline in the ATP stores causes failure of the ionic homeostasis and ultimately infarction. This sequence proposes that acidosis is not result of reduced blood flow (“ischemia” according to the original definition by Rudolf Virchow) or lack of oxygen and anaerobic metabolism. A potential discrepancy between vascular oxygen supply and metabolic oxygen consumption by the myocardial tissue results from catecholamine-mediated excessive oxygen waste.

Under catecholamine influence, cardiac oxygen consumption is excessive; mitochondrial function is impaired. Adrenergic overdrive activates excessive aerobic metabolism that leads to net ATP breakdown with formation of reactive oxygen species, which impair oxidative phosphorylation of ADP. Adrenergic stimulation boosts triglyceride lipolysis and increases levels of free fatty acids. The consequence is accumulation of protons. This acidosis causes reduction in blood flow by impairment of contractility. Decreased contractile output reduces blood flow even more and thus initiates a vicious circle that further increases proton concentration and ultimately results in infarction. To the extent that the excessive aerobic metabolism resulting from adrenergic stimulation comes to a halt the energy deficit can only be compensated for by anaerobic metabolism. Glucose and glycogen become the essential nutrients. As long as the energy provided by anaerobic metabolism keeps the blood flow on a sufficient level, excess protons will be washed out and acidosis and infarction will be prevented. Hence an essential prerequisite for survival of the myocardium in case of excessive adrenergic stimulation is the availability of glucose and glycogen as well as the ability of the heart to utilize these nutrients. Insulin resistance is highly prevalent in the pathogenesis of heart failure. Insulin signalling is essential for normal cardiovascular function, and lack of it results in cardiovascular dysfunction and disease [64].

The outlined sequence describes a pathogenesis of HF and in particular myocardial infarction, which is supported not only by many well-documented experimental findings but as well by extensive clinical experience. Neurohumoral activation as a central detrimental feature of HF is well documented and undisputed [50]. Neurohumoral antagonists have become indispensable in the treatment of HF. Risk factors that are commonly attributed to heart failure like obesity, insulin resistance, critical illness, hypertension, and smoking are all characterized by elevated sympathetic activation. Nicotine causes release of norepinephrine and epinephrine from postganglionic sympathetic nerve endings and the adrenal medulla. Recent epidemiological studies have revealed that smoking restrictions in Germany were followed by reductions in hospitalization for angina pectoris and myocardial infarction [65].

There is a growing awareness that psychosocial factors contribute significantly to the pathogenesis and expression of heart disease. Depression, anxiety, personality factors and character traits, social isolation, and chronic life stress are known to be important risk factors. These psychosocial factors are all associated with sympathetic stimulation [66]. Findings suggest that positive psychological well-being protects consistently against cardiovascular disease, independently of traditional risk factors and ill-being. Specifically, optimism is most robustly associated with a reduced risk of cardiovascular events [67].

A marked elevation in plasma catecholamine levels also underlies stress cardiomyopathy, a syndrome of heart failure that is also known as “tako-tsubo cardiomyopathy”, “transient left ventricular apical ballooning syndrome”, or just “broken heart syndrome” [68]. This syndrome results from emotional, psychological or physical stress, which causes left ventricular dysfunction. It mimics the acute coronary syndrome exhibiting typical features of acute myocardial infarction. However, significant coronary artery disease is invariably excluded. The abnormalities of the left ventricular contraction are transient and the prognosis for complete recovery is good. A report suggests that in stress cardiomyopathy myocardial fatty acid metabolism is more severely impaired than myocardial perfusion [69], indicating that blood flow is sufficient enough to wash out protons generated by the catecholamine-mediated myocardial insult.

If blood flow is restricted through impaired coronary compensatory dilatability or by vascular rigidity and narrowing, oxygen-consuming excessive cardiac sympathetic activity will produce damage of the myocardium. Therefore, atherosclerosis is merely an important risk factor, but not a causal trigger of heart failure and myocardial infarction. The intensity of adrenergic stimulation determines the progression of heart failure - not the extent of atherosclerosis, which can be additionally compensated by collateral blood flow. The eruption of vulnerable plaques is a non-predictable random event that is not causally related to normal progression of angina pectoris and heart failure, which may end in myocardial infarction. This well explains the non-existence of thrombi and occlusions in many instances of myocardial infarction [1, 5].

The pathogenesis of HF outlined above calls for therapies that reduce the catecholamine-induced impairment of the myocardial metabolism that leads to generation of protons and subsequent complications. Beta-adrenergic blockade is used successfully to decrease hyperadrenergic drive. Pharmacological modulation of parasympathetic activity in heart failure is a promising new approach [70]. Vagal stimulation by implanted electrodes in patients with heart failure has been proven to be feasible and safe. Preliminary data suggest that this intervention provides subjective and objective improvements [71]. Investigation of the interactions of hormones that are involved in the autonomous regulation of the heart and modulate myocardial metabolism might be useful to tailor new therapeutic strategies for causal treatment of heart failure.

## Cardiotonic Glycosides Induce Vagus-Mediated Amplification of Insulin Effects

Even as early as the 1930s the German pharmacologist Hans Gremels identified some fundamental correlations between the neurotransmitters of the sympathetic nervous system, epinephrine, and the parasympathetic nervous system, acetylcholine, on myocardial performance and metabolism [72]. Due to the surgical intervention, the heart in a heart-lung preparation is a failing heart. The lifetime of denervated dog heart-lung preparations can be multiplied by infusion of acetylcholine and epinephrine (“humoral innervation”) with simultaneous increase of the efficiency of cardiac workload, indicating that the autonomous nervous system through release of its transmitters is essential for the activity of the heart. Gremels observed that the sympathetic stimulation increases oxygen consumption of the heart, while the parasympathetic activity decreases oxygen consumption. Catecholamines like epinephrine stimulate sympathicotonic dissimilation that is counterbalanced by vagotonic assimilation stimulated by acetylcholine. The vagotonic assimilation regulates the quiescent state of the organism. Through counter regulatory control it is triggered by sympathicotonic activity, which in addition determines its intensity. Gremels thus revealed dynamic interactions of sympathetic and parasympathetic nervous systems that today are known as “accentuated antagonism” [73]. Vagal “tone” (tonic parasympathetic activation) predominates over sympathetic tone at rest. Under normal physiological conditions, parasympathetic stimulation will inhibit tonic sympathetic activation. Elevated sympathetic tone is overridden by intense vagus nerve discharge. Due to this “accentuated antagonism” the effects of catecholamines are very sensitive to changes in concentration. Whereas high concentrations induce increased oxygen consumption, lower concentrations show a decrease in oxygen consumption due to counter regulatory functional activation of the parasympathetic system.

Gremels interpreted increases in oxygen consumption as a consequence of impaired stimulation by acetylcholine and insulin on glucose utilization and glycogen synthesis, which results in a predominance of sympathetically induced oxygen-consuming metabolic activity. He classified such an increase in oxygen consumption as “energetic insufficiency”. Energetic insufficiency always precedes depressed contractile function. Current experiments confirm this observation. Alterations in the myocardial creatine kinase system precede the development of contractile dysfunction in beta(1)-adrenergic receptor transgenic mice [74]. Transgenic overexpression of myofibrillar isoformcreatine kinase in the failing heart of mice significantly increases the rate of in vivo ATP delivery that induces enhanced systolic function, and improves survival [75]. Data from the Studies of Left Ventricular Dysfunction indicate that neurohumoral excitation actually precedes the clinical onset of heart failure [76]. Nevertheless, it still is a widespread assumption that neuro-endocrine activation and its consequences for myocardial metabolism is a reflex to changes in hemodynamics [17]. The experimental and clinical evidence already referred to clearly suggests that autonomic dysregulation and subsequent changes in myocardial metabolism precede heart failure. This is in accordance with the basic laws of physics: any change in contractile output requires preceding provision of energy. First you step on the gas, then the engine accelerates, when you step down from the gas pedal, the engine then slows down, not vice versa.

In Gremels’ dog heart-lung preparations acetylcholine-mediated absorption of glucose from the blood always preceded reduction in oxygen consumption, which was then followed by increase in contractile output. Acetylcholine enhanced the effects of insulin. Addition of glucose, too, increased the effects of acetylcholine, presumably through the stimulation of insulin release. Addition of cardiotonic steroids yielded comparable effects. Therapeutic concentrations of Strophanthus- and Digitalis-glycosides multiplied the effect of acetylcholine dramatically by a factor of 1000. Just like acetylcholine the glycosides first corrected the impaired sugar assimilation, then reduced increased oxygen consumption and finally improved the cardiac output back to normal.

Due to the “accentuated antagonism” low concentrations of epinephrine induce parasympathetic activity by functional activation. Cardiotonic steroids shift the concentrations at which epinephrine still stimulates the vagus to higher levels and thus increase the toxic threshold of sympathetic activation. The potentiation of insulin-like effects of acetylcholine on glucose metabolism by cardiotonic steroids suggests that the action of glycosides ought to be intensified by insulin. Indeed, such an effect was observed in clinical application of Strophanthus glycosides. Concentrated solutions containing as standard 0.25 mg per 0.25 ml for iv-application preferably were diluted with 20 ml of 25% solution of dextrose. This procedure not only reduced the well-known risk of intoxication due to high peak concentration as result of too rapid injection, but also reportedly improved the therapeutic effects [77].

Four decades after Gremels’ experiments, Runge noted [78] that in fact, a tabulation of the therapeutic and toxic effects of cardiac glycosides is strikingly similar to a tabulation of the combined effects of acetylcholine and epinephrine. The results of Gremels’ dog heart-lung experiments have also been confirmed with in-vivo studies in dogs. Digitoxin and k-Strophanthin both show an oxygen sparing effect that precedes hemodynamic alterations [79]. In dogs ouabain induces hypoglycemia. This effect is synergistically enhanced by addition of insulin. Furthermore, ouabain induces increased secretion of insulin and in vitro inhibits the metabolic effects of epinephrine [80]. A clear-cut stimulatory effect of ouabain on the production of insulin in the anesthetized dog has been demonstrated in both infusion and “one shot” experiments [81]. Ouabain promotes the secretion of insulin in pancreatic minces from the toad B. arenarum at non-stimulatory concentrations of glucose and does so at concentrations similar to those of ouabain-like-compounds present in normal human plasma [82]. And ouabain induces increase in the release of acetylcholine from cerebrocortical synaptosomes of rats [83]. Just like insulin, ouabain in picomolar concentration stimulates Na-K-ATPase activity in cultured human renal tubule cells through a Na^+^/H^+^ exchanger-dependent mechanism [84], confirming the insulin-like effects of ouabain.

In Gremels’ interpretation the mode of action of cardiotonic glycosides is primarily the vagus-mediated amplification of insulin effects on glucose absorption and glycogen synthesis that counteracts deleterious effects of excessive sympathetic stimulation. However, in clinical application, digoxin, unlike Strophanthus glycosides has no profound effect on myocardial metabolism. Although all glycosides had similar overall effects, Gremels reports significant differences in onset of action. The effects of the Strophanthus glycoside k-Strophanthin start within minutes, Lanata glycosides (Digilanid A, B and C) have a latency period of 15 - 30 minutes, the effects of the Digitalis glycoside digitoxin only start after 1- 2 hours [85], indicating potential differences in mode of action. Clinical experiences with Strophanthus glycosides document their different therapeutic profile from that of Digitalis glycosides.

## Ouabain is Different From Digitalis Glycosides

Ouabain (referred to as g-Strophanthin in German) and the related Strophanthus glycoside k-Strophanthin have been widely used in Europe, and especially in Germany, to treat heart failure and other forms of disease. In the late 19th century extracts of different origins and different Strophanthus species were used for oral therapy. In 1904 a solution of crystallized ouabain, known as “g-Strophanthin Thoms”, was introduced for oral and iv-application. In 1906 a solution of pure k-Strophanthin with the trade name Kombetin^®^ was introduced for iv-application. Numerous drugs based on ouabain and k-Strophanthin were commercialized. Beginning in the early 1950s different tablet formulations were introduced for oral application of ouabain. Likewise, in the 1950s drugs based on pure digitalis glycosides substituted raw extracts and leaf-based formulations. Digitalis intoxication increased substantially and became a major concern in drug-induced toxicities. These new patent protected medications were promoted with strong marketing efforts. As a consequence, the use of generic Strophanthus glycosides was significantly reduced. Decades of clinical experience disappeared over time. With the introduction of neurohumoral antagonists Strophanthus glycosides eventually fell into oblivion.

The therapeutic profile and the disease profiles for which the use of Strophanthus glycosides is appropriate have been summarized in monographs and reviews [86-89]. Ouabain has a different therapeutic profile from digitalis derivatives. While digitalis is used to treat right ventricular failure, ouabain is used to treat insufficiency of the left ventricle. Ouabain has been used preferentially for the treatment of angina pectoris, including heart attack. Digitalis causes a worsening of symptoms here and is therefore contra-indicated. In addition, ouabain has been used effectively to treat digitalis intoxications; corresponding reports are documented as early as 1902. Recent in-vitro and in-vivo studies confirm this clinical observation [90].

In decades of clinical experience two distinctly different effects of ouabain in treatment of heart disease have been identified: a modest positive inotropic effect, and a stimulating effect on the metabolism of the myocardium. In dogs application of ouabain increases resistance to hypoxia [91, 92] and eliminates cardiac insufficiency induced by ischemia [93]. It is reported that on treatment with ouabain “the animal simply became resistant against O_2_ deficiency for hours”. Hypoxia induced heartache in humans triggered by inhalation of air with low oxygen concentration can be remedied by application of ouabain [77]. The endogenous hormone ouabain, just like the intensively researched phenomenon of ischemic preconditioning, offers multiorgan protection based on innate mechanisms [94].

Today there is much evidence that ouabain is a mammalian hormone produced in the adrenal cortex and hypothalamus. Elevated levels of circulating ouabain have been suggested in chronic renal failure, hyperaldosteronism, congestive heart failure and preeclampsia [95]. Ouabain dose-dependently inhibits the activity of the Na^+^/K^+^-ATPase (NKA). In addition, at low concentrations binding of ouabain to NKA activates multiple signal transduction pathways [95, 96]. Recent research has confirmed the uniqueness of ouabain; supported by the fact that ouabain has a different mechanism of action to digitalis glycosides [97]. Experiments with extremely low concentrations (10^-8^ M) of ouabain in isolated working rat hearts provide strong evidence that the cardioprotective effect of ouabain may be mediated by activation of transduction pathways rather than by inhibition of NKA [98].

## Ouabain is Oats for the Starving Myocardium

Digitalis glycosides induce a positive inotropic effect but do not stimulate myocardial metabolism. While digitalis is likened to a “whip to beat the starving horse”, ouabain has been described as “oats for the starving myocardium”. In dogs, ouabain increases lactate utilisation by the myocardium. Yet, digitoxin not only inhibits lactate utilisation but also induces lactate release [99]. Strophanthus glycosides reduce lactate concentration in the blood of patients with heart diseases [100, 101]. These early reports on effects of Strophanthus glycosides on myocardial metabolism have been repeatedly confirmed. In therapeutic doses ouabain reduces lactate concentration and increases concentration of glycogen. Studies in human skeletal muscle cells indicate that ouabain stimulates glycogen synthesis by activation of GSK3-dependent signalling pathways [102]. Ouabain, when injected intraperitoneally in doses, which were not lethal, induced an increase up to 50-fold in glycogen synthesis in the intact diaphragm of living mice without causing blood sugar alterations [103]. Addition of ouabain not only preserved high-energy phosphates and glycogen, but also improved the post-ischemic function of rabbit hearts stored ex vivo [104].

In contrast, digitalis derivatives do not exert the metabolic effects of ouabain on the heart. Digitoxin inhibits glucose induced insulin release in the perifused islets of the rat [105]. Digoxin caused a decrease of cardiac glycogen when given to rats [106]. Lanatoside-C has a negative effect on carbohydrate metabolism in the intact human heart [107]. Non-toxic doses of digitoxin increase the rate of turnover of high-energy phosphate and of glycogen in dog hearts [108]. Thus the metabolic effects suggest that, unlike ouabain, digitalis glycosides stimulate the sympathetic system.

The clinical benefits of digoxin are based on a positive inotropic effect, which is commonly attributed to partial inhibition of NKA. This effect of digoxin in intact animals and in human beings is quite small and often difficult to demonstrate. Although questioned by contradictory findings [109], some evidence indicates improvement of baroreceptor function by digoxin, decreased sympathetic tone, and increased parasympathetic tone, favourably influencing autonomic balance in heart failure [110]. However, experimental evidence documents sympathomimetic action of digitalis [111]. These sympathomimetic effects have been mostly neglected in the clinical literature.

Just as with catecholamines, the effects of digoxin are highly dose dependent. Only low serum digoxin levels (i.e. 0.5 - 0.8 ng/ml) correlate with a favourable treatment effect in heart failure. The presented evidence suggests that, just like low doses of catecholamines, these low doses of digoxin cause decreased sympathetic tone and increased parasympathetic tone due to counter regulatory functional activation of the parasympathetic system (“accentuated antagonism”). Even slightly higher concentrations result in sympathetic stimulation ending in digoxin-intoxications. This well explains clinical experiences where ouabain has been used preferentially for the treatment of angina pectoris, including heart attack, while digoxin causes a worsening of symptoms here and is therefore contra-indicated. This interpretation also is in agreement with the cited experimental and clinical observations that ouabain can act as an antidote for digitalis intoxication.

The metabolic effects of ouabain indicate that ouabain, unlike digoxin, possesses intrinsic sympatholytic properties. At the extremely low concentration of 10^-10^ Mol/L ouabain caused a significant inhibition of spontaneous and of acetylcholine-induced release of catecholamines from adrenal medulla of rats [112]. In addition, there is experimental evidence that therapeutic doses of ouabain substantially reduce concentrations of noradrenalin in the urine of healthy volunteers [113]. In contrast, digoxin treatment yields no significant changes in plasma catecholamines of healthy subjects [114]. In patients with severe congestive heart failure high levels of norepinephrine were not altered by treatment with digoxin [115]. In a double-blind crossover evaluation of k-strophanthin versus digoxin in patients with advanced congestive heart failure due to dilated cardiomyopathy patients had an almost threefold rise from normal of circulating norepinephrine, which was not affected by digoxin but was reduced by almost 50% by k-strophanthin [116].

Despite centuries of successful clinical use, controlled clinical studies of digitalis glycosides have shown ambivalent results. The Digitalis Investigation Group trial has indicated that digoxin is quite effective in reducing cardiovascular hospitalisations but failed to improve mortality rates. A more recent prospective study over 8 years on 4467 patients with HF suggests that therapy with digoxin is associated with an improved mortality and morbidity of HF, including women and patients with non-systolic HF [117]. There is still serious clinical interest in this drug. Digoxin therapy has a major argument in its favour: a low price with an extremely positive cost-effectiveness ratio. But the main obstacle that limits the therapeutic application of digoxin is a very narrow therapeutic index. Gremels has shown that the effect of digitalis is very dependent on the status of the autonomic nervous system. At low doses the accentuated antagonism of the autonomic nervous system converts the inherent sympathomimetic effect of digoxin into a beneficial parasympathetic effect for the heart. In higher doses the stimulation of the sympathetic system prevails. This constitutes the sensitive dose-dependence of digoxin effects, which is the underlying explanation for the ambiguous clinical findings.

Further clinical experience illustrate that ouabain prevents the detrimental effects of sympathetic over-stimulation. In cardiac surgery, strict pH control is imperative. In the “Strophanthin era”, German surgeons routinely applied 0.3 mg of Strophanthus glycosides pre-operatively and thereby observed significantly fewer complications [86]. Von Ardenne demonstrated that in myocardial infarction induced by ligature in rat and rabbit hearts, the pH in myocardial tissue drops markedly. Administration of ouabain raises the pH of acidic cardiac tissue within a few minutes by up to 0.5 units [118]. Digitoxin does not alter the pH. The toxicity of ouabain depends on the form of application. In guinea pigs the 24-hour LD50 for oral administration has been determined as 46.4 mg/kg and for iv-administration as 0.168 mg/kg [119]. These toxicity data indicate a wide and adequately safe therapeutic index for oral administration.

The mechanism of action of digitalis glycosides for decades has been subject of intensive research. This research was guided by the assumption that all cardiotonic glycosides have identical mode of action. In addition, the modulation of the autonomic nervous system by the cardiotonic glycosides and the decisive influence of the accentuated antagonism on myocardial metabolism have hitherto been carelessly neglected. The demonstration that ouabain specifically inhibits the Na^+^/K^+^-ATPase (NKA) transformed ouabain into a crucial tool for studying the intrinsic mechanisms of this membrane enzyme. Today it is presented as standard knowledge in textbooks that the positive inotropic effect of cardiotonic steroids is due to partial inhibition of NKA. However, the drug concentrations applied in clinical application result in plasma concentrations of cardiac glycosides that are far below the concentration needed to cause an inhibition of NKA. Ouabain exerts its therapeutic effects already in low nanomolar and even picomolar concentrations. In addition, a critical analysis of the available data proposes that Strophanthus glycosides have a distinct different mode of action than Digitalis glycosides [97]. Digitalis glycosides mimic the effects of catecholamines, whereas Strophanthus glycosides counteract sympathetic activity. These in principle opposing effects at low concentrations of digitalis are disguised by the accentuated antagonism, which at low concentrations of glycosides forces the opposing effects into line.

## Conclusion

The standard textbook explanation for heart failure proposes that this disease state and myocardial infarction are caused by an imbalance between myocardial oxygen supply and myocardial oxygen demand due to impaired blood flow to the heart. An increasing body of clinical observations and experimental evidence reinforces doubt about this model. There is a wealth of evidence suggesting that cardiac dysfunction results from autonomic dysregulation of the contractile output of the heart. Excessive activation of the sympathetic nervous system and a decrease in parasympathetic tone are associated with increased mortality. Circulating catecholamine levels closely correlate with the severity and poor prognosis in heart failure and thus play a critical role in the development of cardiovascular diseases. Catecholamines at low concentrations are beneficial in regulating heart function by exerting a positive inotropic action on the myocardium, whereas high concentrations of catecholamines produce deleterious effects on the cardiovascular system. Decreased cholinergic neurotransmission causes alterations that contribute to heart dysfunction.

Any change in contractile output of the heart is consequence of altered myocardial metabolism. Sympathetic over-stimulation causes increased levels of catecholamines, which induce excessive aerobic metabolism leading to excessive cardiac oxygen consumption. Resulting impaired mitochondrial function causes acidosis, which results in reduction in blood flow by impairment of contractility. To the extent that the excessive aerobic metabolism resulting from adrenergic stimulation comes to a halt, the energy deficit has to be compensated for by anaerobic metabolism. Glucose and glycogen become the essential nutrients. As long as the energy provided by anaerobic metabolism keeps the blood flow on a sufficient level, acidosis and infarction will be prevented.

Neurohumoral antagonists block adrenergic over-stimulation but do not provide the heart with fuel for compensatory anaerobic metabolism. The endogenous hormone ouabain reduces catecholamine concentration in healthy volunteers, promotes the secretion of insulin, induces release of acetylcholine from synaptosomes and potentiates the stimulation of glucose metabolism by insulin and acetylcholine. Ouabain stimulates glycogen synthesis and increases lactate utilisation by the myocardium. Decades of clinical experience with ouabain confirm the cardioprotective effects of this endogenous hormone, which effectively prevents myocardial infarction. In addition, ouabain has been used in treatment of diseases that are also known to be related to deleterious over-stimulation of the sympathetic system. Clinical experience reports successful application of ouabain in treatment of stroke [120] and shock [121]. In patients with the syndrome of cerebral malnutrition, ouabain therapy has proven very effective [122]. This knowledge has been lost over time. The so far neglected sympatholytic and vagotonic effects of ouabain on myocardial metabolism clearly make a clinical re-evaluation of this endogenous hormone necessary. Clinical studies with ouabain that correspond to current standards are warranted.
